# Survival and mortality of elderly men with localized prostate cancer managed with primary androgen deprivation therapy or by primary observation

**DOI:** 10.1186/s12894-020-00593-7

**Published:** 2020-03-12

**Authors:** Heikki Seikkula, Peter J. Boström, Karri Seppä, Janne Pitkäniemi, Nea Malila, Antti Kaipia

**Affiliations:** 1 Department of Surgery, Central Hospital of Central Finland, Jyväskylä, Finland; 2 Department of Urology, Turku University Hospital, Turku, Finland; 3 Department of Urology, University of Turku, Turku, Finland; 4 Finnish Cancer Registry, Helsinki, Finland; 5 Faculty of Social Sciences (Health Sciences), Tampere, Finland; 6 Department of Public Health, University of Helsinki, Helsinki, Finland; 7 School of Health Sciences, University of Tampere, Tampere, Finland; 8 Department of Urology, Tampere University Hospital, Tampere, Finland

**Keywords:** Prostate cancer survival, Prostate cancer–specific mortality, Localized prostate cancer, Androgen deprivation therapy

## Abstract

**Background:**

Androgen deprivation therapy (ADT) remains a primary treatment for localized prostate cancer (PCa) even though there is no evidence that its use is beneficial in the absence of curative treatment.

**Methods:**

Men aged ≥70 years (*n* = 16,534) diagnosed with localized PCa from 1985 to 2014 and managed either with primary observation or ADT in the absence of curative treatment were included. The cases were identified from the population-based Finnish Cancer Registry. We estimated the standardized mortality ratios (SMR) for overall mortality by treatment group. We determined the relative risk (RR) of PCa-specific mortality (PCSM) and other-cause mortality between the two treatment groups. Survival was determined using the life table method. Two age groups (70–79 years and ≥ 80 years) and three calendar time cohorts (1985–1994, 1995–2004, and 2005–2014) were compared following adjustment of propensity score matching between the treatment groups with four covariates (age, year of diagnosis, educational level, and hospital district). Follow-up continued until death or until December 31, 2015.

**Results:**

Patients in the observation group had lower overall SMRs than those in the ADT group in both age cohorts over the entire study period. PCSM was higher in men aged 70–79 years undergoing primary ADT compared to those managed by observation only (RR: 1.70, 95% confidence interval [CI]: 1.29–2.23 [1985–1994]; RR 1.55, 95% CI: 1.35–1.84 [1995–2004]; and RR 2.71, 95% CI: 2.08–3.53 [2005–2014]); *p* = 0.005 for periodic trend. A similar trend over time was also observed in men aged > 80 years; (*p* for age–period interaction = 0.237). Overall survival was also higher among men in their 70’s managed by observation compared to those undergoing ADT.

**Conclusions:**

Primary ADT within four months period from diagnosis is not associated with improved long-term overall survival or decreased PCSM compared to primary conservative management for men with localized PCa. However, this observational study’s conclusions should be weighted with confounding factors related to cancer aggressiveness and comorbidities.

## Background

Androgen deprivation therapy (ADT) has been the cornerstone of treatment for locally advanced and metastatic (M+) prostate cancer (PCa) since the 1940s [1]. Immediate ADT or ADT combined with docetaxel or abiraterone acetate is the current treatment of choice for M+ PCa [2]. However, the use of ADT increased sharply between 1989 and 2001 in the USA despite the fact that ≤5% of patients with newly diagnosed PCa have distant metastases at first presentation compared with 20–25% ≥20 years ago [3, 4]. While the increased use of ADT is partly accounted for by the uptake of neoadjuvant and adjuvant treatment along with radiation therapy, it is primarily elucidated by ADT for localized disease, especially for elderly patients [5]. Thus, ADT is commonly used to treat localized PCa although it has not been shown to improve survival [6].

The risk of metastases or death from conservatively managed clinical stage T1/T2 cancers was estimated in a meta-analysis of six studies from the era prior to prostate-specific antigen (PSA) [7]. The risk of metastasis at 10 years was found to be 19, 42, and 74% for well-differentiated, moderately differentiated, and poorly differentiated tumors, respectively [7]. Similarly, the long-term clinical outcomes of localized PCa without initial treatment with curative intent during the PSA era were assessed [8]. Thirty per cent of the patients died of PCa and 30% of other causes within a 12-year period [8]. A landmark Swedish study demonstrated a benign course of well- or intermediately differentiated PCa in the absence of initial treatment with curative intent [9]. Thus, the clinical course of localized high-risk PCa can be progressive, but the majority of cancers are indolent and slow to progress [2]. According to current guidelines, observation with the option of later treatment in the case of disease progression (i.e., watchful waiting) is recommended for localized and locally advanced PCa in elderly patients with competing comorbidities.

The objective of this observational study was to investigate mortality in elderly PCa patients primarily managed with ADT or observation only during long follow-ups in Finland.

## Methods

### Study population

The Finnish Cancer Registry is a nationwide population-based register of all incident cancer cases diagnosed in Finland since 1953. The health care personnel in hospitals, outpatient clinics, and healthcare facilities are obligated to notify of new cases. Additionally, pathology notifications are received from all histopathological laboratories in Finland. The registry coverage is estimated at 99% for male genital cancers [10]. The spreading into localized, locally advanced, or metastatic are classified by tumor size, regional node involvement, and presence of metastasis for cases covered by the Finnish Cancer Registry.

The Finnish Cancer Registry data can be linked with the population register center database for dates of death or emigration and causes of death and education levels from Statistics Finland.

We identified all PCa patients aged 70 years or older at diagnosis with localized cancer (clinical stage T1/T2) from 1985 to 2014 and managed by primary ADT or observation in the absence of radical treatment with curative intent (Table 1). Of the total patients identified (*n* = 16,534), 11,572 were aged 70–79 years and 4962 were ≥ 80. Within four months of diagnosis, the primary treatment was ADT (ADT group *n* = 9704), while 6830 received no treatment (observation group) (Table 1). The regional data included 22 hospital districts taking care of specialized care. The survival rates of the patients were compared by age and treatment group over three periods (1985–1994, 1995–2004, and 2005–2014).

**Table 1:** Study population of prostate cancer patients

	All patients			
	ADT		Observation	
Variable	N	%	N	%
**Age at diagnosis**				
70–74	3096	31.9	2850	41.7
75–79	3411	35.2	2215	32.4
80–84	2170	22.4	1227	18.0
85–89	832	8.6	438	6.4
> 90	195	2.0	100	1.5
Total	9704	100	6830	100
**Year of diagnosis**				
1985–1989	785	8.1	208	3.0
1990–1994	1197	12.3	337	4.9
1995–1999	1724	17.8	797	11.7
2000–2004	2159	22.2	1313	19.2
2005–2009	2280	23.5	2194	32.1
2010–2014	1559	16.1	1981	29.0
Total	9704	100	6830	100
**Education level**				
Basic	6920	71.3	4421	64.7
Secondary	1325	13.7	1074	15.7
High	1459	15.0	1335	19.5
Total	9704	100	6830	100
**Hospital district**				
Uusimaa	932	9.6	786	11.5
Helsinki	812	8.4	698	10.2
Varsinais-Suomi	835	8.6	628	9.2
Satakunta	500	5.2	281	4.1
Kanta-Hame	471	4.9	322	4.7
Pirkanmaa	1187	12.2	614	9.0
Paijat-Hame	668	6.9	304	4.5
Kymenlaakso	433	4.5	182	2.7
Etela-Karjala	321	3.3	179	2.6
Etela-Savo	158	1.6	102	1.5
Ita-Savo	76	0.8	64	0.9
Pohjois-Karjala	149	1.5	178	2.6
Pohjois-Savo	466	4.8	403	5.9
Keski-Suomi	374	3.9	557	8.2
Etela-Pohjanmaa	522	5.4	386	5.7
Vaasa	285	2.9	182	2.7
Keski-Pohjanmaa	136	1.4	128	1.9
Pohjois-Pohjanmaa	610	6.3	372	5.4
Kainuu	265	2.7	99	1.4
Lansi-Pohja	280	2.9	132	1.9
Lappi	190	2.0	177	2.6
Åland	34	0.4	56	0.8
Total	9704	100	6830	100

*ADT* androgen deprivation therapy, *N* number

### Statistical analysis

Overall survival was evaluated using the life table method [11]. The Poisson regression model was used to quantify differences in patient mortality between the defined groups. The results were reported as relative risk (RR) of PCa-specific mortality (PCSM) and mortality due to causes other than PCa. We also estimated the standardized mortality ratio (SMR) for overall mortality among the patients. The SMR is estimated as the ratio of observed and expected numbers of deaths. The latter was derived from the mortality rates of the male population in Finland stratified by age (1-year intervals), calendar year and education. We used a nearest neighbor matching with a logistic regression–based propensity score [12] to identify a cohort of 5715 paired patients. In the logistic regression, the probability of a given treatment was modeled as a function of age and calendar period (in 5-year groups), education, and hospital district. Interactions between calendar period and each of the covariates were also included in the model. Statistical analysis was performed with R (version 3.2.3) using the packages popEpi [13] and MatchIt [14].

The study protocol was approved by the institutional review board of the Hospital District of Southwest Finland. The National Institute for Health and Welfare (Finland) approved access to the registry data (study number 182/5.05.00/2015). Statistics Finland approved access to the data on the cause of death (study number TK-53-86-17).

## Results

The stage distribution of all prostate cancer patients from 1985 to 2014 (*n* = 95,959) in Finland is shown in Supplementary Table 1. Approximately half of the patients were classified as having localized PCa. The proportion of metastatic disease decreased from 28 to 17% between the periods 1985–1994 and 2005–2014. However, the PCa stage was missing in nearly one-third of the patients over the study period (Supplementary Table 1). In this study, we included only PCa patients aged 70 years or older at diagnosis who received primary treatment with ADT or no treatment within four months of diagnosis (Table 1).

SMR analysis showed that overall mortality was lower in the observation group than in the ADT group in all three time periods. However, a declining trend in SMR over time was seen in both groups. Furthermore, over the most recent period (2005–2014), patients in both age cohorts had lower overall mortality than the general male population in Finland (SMR: 0.93, 95% CI: 0.88–0.98) (Table 2). Propensity score matching did not change any of these results.

**Table 2:** Overall mortality of the study patients compared to that of the male population of Finland

All patients	Period					
	1985–1994		1995–2004		2005–2014	
	ADT	Observation	ADT	Observation	ADT	Observation
Age	SMR (95% confidence interval)					
All (> 70)	1.48 (1.41–1.54)	1.20 (1.10–1.30)	1.19 (1.15–1.23)	1.03 (0.98–1.08)	1.08 (1.03–1.13)	0.93 (0.88–0.98)
70–79	1.57 (1.48–1.65)	1.17 (1.05–1.30)	1.24 (1.19–1.29)	1.05 (0.99–1.11)	1.11 (1.04–1.19)	0.95 (0.88–1.01)
≥80	1.39 (1.22–1.42)	1.25 (1.08–1.43)	1.10 (1.04–1.17)	1.00 (0.92–1.09)	1.04 (0.97–1.11)	0.89 (0.82–0.97)
Matched pairs						
All (> 70)	1.50 (1.38–1.63)	1.19 (1.09–1.30)	1.19 (1.13–1.25)	1.03 (0.98–1.08)	1.09 (1.03–1.15)	0.93 (0.87–0.98)
70–79	1.57 (1.41–1.74)	1.16 (1.04–1.29)	1.23 (1.16–1.30)	1.05 (0.99–1.11)	1.12 (1.05–1.20)	0.94 (0.87–1.01)
≥ 80	1.39 (1.21–1.60)	1.25 (1.08–1.44)	1.12 (1.02–1.21)	1.00 (0.91–1.08)	1.03 (0.94–1.12)	0.91 (0.82–0.99)

Population mortality stratified by age and calendar year and education level

*ADT* androgen deprivation therapy, *SMR* standardized mortality ratio

In both age cohorts, we observed higher PCSM in patients undergoing primary ADT compared to those managed by observation only (Table 3). The relative risk of prostate cancer mortality increased significantly over time in the ADT group compared to the observation group (*p* for trend = 0.05). On the other hand, the risk of death from causes other than PCa decreased over time in patients aged 70–79 years undergoing primary ADT compared to those managed by observation only, but the same was not true in patients aged 80 years or older (Table 3).

**Table 3:** Risk of PCa and other-cause mortality among men on primary ADT compared to observation only

Time period (age)	Relative risk of PCa mortality (95% CI)		Relative risk of mortality due to causes other than PCa (95% CI)	
	ADT	Observation	ADT	Observation
1985–1994 **(70–79y)**	1.70 (1.29–2.23)	1.00 (ref)	1.22 (1.00–1.49)	1.00 (ref)
1995–2004	1.55 (1.31–1.84)	1.00 (ref)	1.07 (0.97–1.18)	1.00 (ref)
2005–2014	2.71 (2.08–3.53)	1.00 (ref)	1.03 (0.92–1.16)	1.00 (ref)
1985–1994 **(>80y)**	1.30 (0.89–1.88)	1.00 (ref)	1.09 (0.85–1.39)	1.00 (ref)
1995–2004	1.49 (1.13–1.97)	1.00 (ref)	1.05 (0.92–1.20)	1.00 (ref)
2005–2014	1.65 (1.19–2.29)	1.00 (ref)	1.06 (0.91–1.23)	1.00 (ref)

*p*-value for age–period interaction = 0.237; *p* for age effect = 0.052; *p* for period effect = 0.005. Analysis is based on propensity matched pairs

*PCa* prostate cancer, *CI* confidence interval, *ADT* androgen deprivation therapy, *ref*. reference

**Fig. 1:**
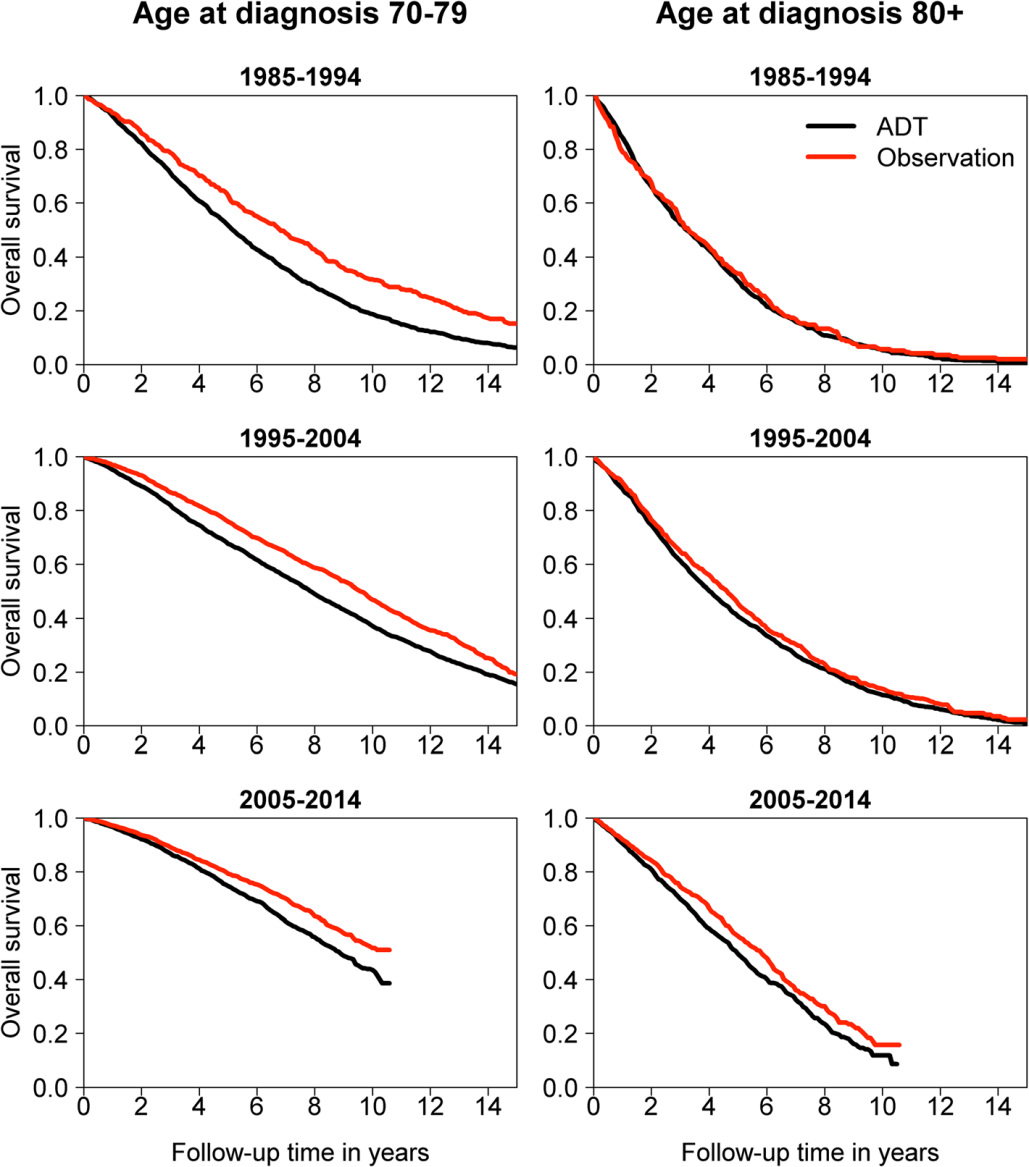
Overall survival among men aged 70–79 years and among men aged 80 or older

Overall survival of PCa patients aged 70–79 years was higher in the observation group than in the ADT group throughout the study period. However, a rising trend was observed in both treatment groups. Among patients over 80 years of age, overall survival improved over time in both treatment groups with no significant differences between them until the most recent period (from 2005 to 2014), when overall survival in the observation group was higher than in the ADT group (Fig. 1). The results remained similar after propensity score matching.

## Discussion

In this population-based study, we investigated whether the survival and mortality outcomes in elderly male patients (aged ≥70 years) with localized PCa is different between those managed by primary observation and those undergoing primary ADT over a long period of time. We showed that the overall SMR of patients in the observation cohort was lower than those in the ADT cohort in both age groups over the entire study period. PCSM and mortality due to causes other than PCa were lower in patients monitored with primary observation compared to those managed with primary ADT. This difference was particularly evident in patients aged 70–79 years at the time of diagnosis. Overall survival was also higher in subjects in this age group managed by primary observation. By contrast, a smaller difference in PSCM was observed between patients undergoing ADT and those managed by primary observation, while a clear difference in mortality due to other causes was not seen in patients aged 80 or older. This implies a better general health condition and a healthier lifestyle among patients in the observation cohort.

An increased risk of PCSM was observed in patients undergoing primary ADT compared to those managed by primary observation, while a lower risk of other-cause mortality was seen in the younger patient group managed by primary observation. This suggests that in recent times, primary ADT without curative treatment was generally selected for patients with aggressive disease. A 10-year threefold risk of PCSM was associated with poorly differentiated PCa among patients with conservatively managed localized PCa in the Surveillance, Epidemiology, and End Results (SEER) Program [15]. Most male patients with conservatively managed localized PCa aged > 66 years with competing comorbidities died from causes other than PCa in a period of 10 years, irrespective of age and tumor aggressiveness [16]. The use of primary ADT was beneficial for patients with aggressive disease and few comorbidities [16]. However, overall mortality was lower in subjects managed by observation than with ADT. We assumed that a shift from ADT to a more radical treatment may have occurred in patients with a good general health status. Since an increasing number of PCa cases has been diagnosed in recent years, and observation is widely utilized in Finland, it is likely that observation was selected for patients with less aggressive histology and without advanced disease. Consequently, fewer male patients died of cancer owing to the slow, natural course of disease.

Overall survival was reduced in patients aged 70–79 years initially treated with. It is still difficult to draw a conclusion from this finding owing to the absence of data on patient comorbidities. However, increased morbidity and mortality from ADT-related side effects is possible. Thus, the survival benefits of ADT are partly offset by its high toxicity. These findings can also be explained by a better general health condition among patients in the observation group. In our cohort, patients in the observation group had a clearly lower risk of overall mortality compared to patients undergoing primary ADT. Since in epidemiological studies the only endpoint that is free from bias is mortality, we can assume that patients in observation cohort were healthier than those in the ADT cohort.

Many population-based analyses suggest that gonadotropin-releasing hormone (GnRH) agonist use is associated with a greater risk of coronary artery disease, myocardial infarction, and diabetes mellitus (DM) [17–19]. Subsequent reports have suggested that male patients with comorbidities or prior cardiovascular disease treated with GnRH agonists might be at increased risk of cardiovascular mortality [20, 21]. Based on these observations, a science advisory consensus statement on GnRH agonist therapy and cardiovascular risk was issued, together with a U.S. Food and Drug Administration safety warning to address concerns of increased risk of myocardial infarction, stroke, sudden cardiac death, and DM [22]. However, conflicting results have been reported. In a recent meta-analysis, ADT use was not associated with an increased risk of cardiovascular death, but with a lower risk of PCSM and all-cause mortality [23]. Studer et al. also reported that ADT for localized PCa may even reduce cardiovascular mortality if started immediately after diagnosis [24].

The current study findings did not support the life-prolonging effects of primary ADT for localized PCa. Several reports have shown similar results: In a prior population-based cohort study on 66,717 Medicare patients diagnosed between 1992 and 2009, and who received no definitive local therapy within 180 days of prostate cancer diagnosis, primary ADT was not associated with improved overall long-term CSS or the CSS of patients with localized PCa [25]. Instead, there is evidence that primary ADT led to inferior outcomes [24]. Low overall survival rates were reported for male patients with localized disease treated with primary ADT rather than observation in a previous population-based study [26]. In addition, Potosky et al. reported that primary ADT was neither associated with an enhanced risk of all-cause mortality (hazard ratio [HR] of 1.04, 95% CI: 0.97–1.11) nor PCa-specific mortality (HR of 1.03; 95% CI: 0.89–1.19) after adjusting for the sociodemographic and clinical characteristics of patients with localized PCa. However, primary ADT was associated with a decreased risk of all-cause mortality, but not PCSM, among patients at high risk of PCa progression [27].

PSA screening practices have increased exponentially over a 30-year period, and regular PSA testing is used frequently among all socioeconomic groups in Scandinavia and Finland [28, 29]. In the early years of the present study, a diagnosis of localized PCa was generally performed via a digital rectal examination or using pathological specimens obtained following a transurethral resection of the prostate. In recent years, most localized PCa cases have been diagnosed by prostate biopsies prompted by elevated PSA values. Moreover, although a proven benefit of the PSA screening of older males has not been shown, PSA testing is frequently performed for elderly patients [30]. The wide-stage migration of PCa from advanced to indolent disease has been reported over ≥20 years [31]. Thus, a commonly employed but poorly organized screening policy explains the increased rate of PCa in past decades in Finland. Consequently, the number of elderly male patients with PCa has also increased. This implies that the more favorable outcomes associated with the use of primary observation compared to ADT in patients with PCa, especially those in their 70s, can be attributed to PSA-related “lead time” rather than life extension.

Over the study period, staging of metastatic disease in Finland was performed by bone scan examination according to national guidelines until the mid-2000s, after which low-risk PCa patients typically underwent no imaging. The proportion of de novo metastatic PCa in Finland was nearly one-third from 1985 to 1994. This implies that staging procedures of PCa by bone scans were widely performed even in the earlier years of the study period, suggesting a good quality of the TNM classification data of PCa in the Finnish Cancer Registry database. In the Finnish Cancer Registry reports, the proportion of de novo metastatic PCa has recently been around 17%, higher than in Sweden [32], where the incidence of PCa is high and PSA screening is widely performed. This might also indicate a more aggressive PCa histology in cases that are classified as localized by the TNM system, and thus more commonly treated with immediate ADT than cases with less aggressive histology.

A few points are worthy of further consideration. As information on patient comorbidities and detailed PCa characteristics (i.e., Gleason scores and PSA values) was not collected, it was not possible to adjust for differences in morbidity and mortality with a potential link to ADT. Thus, a comparison of study outcomes in terms of PCSM and overall survival between the different groups was not possible. Also, due to the absence of data on patient comorbidities and PCa characteristics, it was not possible to determine who would benefit from primary ADT in localized PCa. However, propensity score matching, particularly when applied to socioeconomic status/education level, may have mitigated some of the comorbidity-related limitations. Detailed information on cancer treatment was also incomplete for both treatment groups. Although men in the observation group did not receive ADT or radical treatment in the four months following diagnosis, it is conceivable that some of them may have received treatment later. This limitation may have had a substantial effect on the study outcomes. Furthermore, although the study population included patients with localized PCa, the reliability of the staging procedures over time is debatable. TNM staging was based on mandatory reports obtained from hospitals and pathological laboratories. In other words, clinical practices in Finland were governed by national and/or European prostate cancer guidelines of the time. Consequently, the risk of metastases was evaluated according to these guidelines.

Notwithstanding these limitations, the study had several strengths. It was an observational population-based study estimating trends in cancer survival and mortality over time. While we did not directly compare different types of cancer treatments, population-based cohorts yield important information about PCa treatment in real-life situations, as the populations in randomized controlled trials are selected using stringent criteria by excluding out substantial proportion of real-life patients. However, in population-based studies patients are also allocated to different treatments, and results based on those can be subject to errors when comorbidities are involved. From an epidemiological perspective, these data determine the Finnish results obtained for patients managed with primary ADT or by observation. This study was based on nationwide data for PCa and included nearly 100% of patients in Finland over a nearly 30-year period. No previous population-based results from Finland on this subject have been published. Similar complete population-based coverage is not available in many European countries.

## Conclusion

Primary ADT within four months period from diagnosis is not associated with improved long-term overall survival or decreased PCSM than primary conservative management for men with localized PCa. However, the study’s results may have been affected by patient selection or treatment intention as a consequence of general health and healthcare service–related factors.

## Supplementary information

**Additional file 1: Table S1.** Stage distribution of prostate cancer patients by TNM-classification in Finland from 1985 to 2014.

## Data Availability

The datasets used and/or analyzed during the current study are available from the corresponding author upon reasonable request.

## Abbreviations

ADT: androgen deprivation therapy
PCa: prostate cancer
PCSM: prostate cancer–specific mortality
RR: relative risk
SMR: standardized mortality ratio
