# Supplementary material for: Survival and mortality of elderly men with localized prostate cancer managed with primary androgen deprivation therapy or by primary observation
Source: BMC Urol. 2020 Mar 12;20:25. doi: 10.1186/s12894-020-00593-7 (PMC7069023; doi:10.1186/s12894-020-00593-7)
Supplement: Supplementary file 1 — Additional file 1: Table S1. Stage distribution of prostate cancer patients by TNM-classification in Finland from 1985 to 2014. [file 12894_2020_593_MOESM1_ESM.docx]

| Age | Stage | Number of patients (%) | | | |
| --- | --- | --- | --- | --- | --- |
|  |  | Year of prostate cancer diagnosis | | | |
|  |  | 1985-2014 | 1985-1994 | 1995-2004 | 2005-2014 |
| All age-groups | Localized | 47107 (49.1) | 6636 (44.8) | 16632 (48.2) | 23839 (51.1) |
|  | Locally Advanced | 2285 (2.4) | 8 (0.1) | 280 (0.8) | 1997 (4.3) |
|  | Local node positive | 877 (0.9) | 229 (1.5) | 318 (0.9) | 330 (0.7) |
|  | Metastatic | 18250 (19.0) | 4170 (28.2) | 6116 (17.7) | 7964 (17.1) |
|  | Unknown | 27440 (28.6) | 3763 (25.4) | 11154 (32.3) | 12523 (26.8) |
|  | Total | 95959 (100.0) | 14806 (100.0) | 34500 (100.0) | 46653 (100.0) |
| <70 years | Localized | 23855 (55.8) | 2177 (46.3) | 8341 (55.9) | 13337 (57.6) |
|  | Locally Advanced | 1271 (3.0) | 6 (0.1) | 165 (1.1) | 1100 (4.7) |
|  | Local node positive | 575 (1.3) | 113 (2.4) | 232 (1.6) | 230 (1.0) |
|  | Metastatic | 7182 (16.8) | 1485 (31.6) | 2324 (15.6) | 3373 (14.6) |
|  | Unknown | 9874 (23.1) | 917 (19.6) | 3849 (25.8) | 5108(22.1) |
|  | Total | 42757 (100.0) | 4698 (100.0) | 14911 (100.0) | 23148 (100.0) |
| 70-79 years | Localized | 16947 (46.7) | 3024 (46.0) | 6075 (44.8) | 7848 (48.6) |
|  | Locally Advanced | 706 (2.0) | 1 (0.0) | 100 (0.7) | 605 (3.8) |
|  | Local node positive | 234 (0.6) | 89 (1.4) | 63( 0.5) | 82(0.5) |
|  | Metastatic | 7009 (19.3) | 1683 (25.6) | 2480 (18.3) | 2846 (17.6) |
|  | Unknown | 11372 (31.4) | 1772 (27.0) | 4833 (35.7) | 4767(29.5) |
|  | Total | 36268 (100.0) | 6589 (100.0) | 13551 (100.0) | 16148 (100.0) |
| >80 years | Localized | 6305 (37.2) | 1435 (40.6) | 2216 (36.7) | 2654 (36.1) |
|  | Locally Advanced | 308 (1.8) | 1 (0.0) | 15 (0.2) | 292 (4.0) |
|  | Local node positive | 68 (0.4) | 27 (0.8) | 23 (0.4) | 18(0.2) |
|  | Metastatic | 4059 (24.0) | 1002 (28.3) | 1312(21.7) | 1745(23.7) |
|  | Unknown | 6194( 36.6) | 1074 (30.3) | 2472 (41.0) | 2648 (36.0) |
|  | Total | 16934 (100.0) | 3539 (100.0) | 6038 (100.0) | 7357 (100.0) |

Supplementary table 1. Stage distribution of prostate cancer patients by TNM-classification in Finland from 1985 to 2014.
